# Identifying Chemical Groups for Biomonitoring

**DOI:** 10.1289/EHP537

**Published:** 2016-12-01

**Authors:** Gail Krowech, Sara Hoover, Laurel Plummer, Martha Sandy, Lauren Zeise, Gina Solomon

**Affiliations:** 1 Office of Environmental Health Hazard Assessment, Oakland, California, USA; 2 California Environmental Protection Agency, Sacramento, California, USA

## Abstract

Regulatory agencies face daunting challenges identifying emerging chemical hazards because of the large number of chemicals in commerce and limited data on exposure and toxicology. Evaluating one chemical at a time is inefficient and can lead to replacement with uncharacterized chemicals or chemicals with structural features already linked to toxicity. The Office of Environmental Health Hazard Assessment (OEHHA) has developed a process for constructing and assessing chemical groups for potential biomonitoring in California. We screen for chemicals with significant exposure potential and propose possible chemical groups, based on structure and function. To support formal consideration of these groups by Biomonitoring California’s Scientific Guidance Panel, we conduct a detailed review of exposure and toxicity data and examine the likelihood of detection in biological samples. To date, 12 chemical groups have been constructed and added to the pool of chemicals that can be selected for Biomonitoring California studies, including p,p´-bisphenols, brominated and chlorinated organic compounds used as flame retardants, non-halogenated aromatic phosphates, and synthetic polycyclic musks. Evaluating chemical groups, rather than individual chemicals, is an efficient way to respond to shifts in chemical use and the emergence of new chemicals. This strategy can enable earlier identification of important chemicals for monitoring and intervention.

## Introduction

Environmental health researchers and government scientists in the United States typically encounter a paucity of information on both toxicity and exposure for most chemicals in commerce (Judson et al. 2009; Egeghy et al. 2012; Neltner et al. 2013; Silbergeld et al. 2015). Even if data are available, federal and state agencies have limited capacity for assessing health risks (GAO 2013). The recent legislative reform to the Toxic Substances Control Act (TSCA) includes provisions that aim to increase data availability for chemicals in commerce and expand safety reviews by the U.S. Environmental Protection Agency (EPA) (Frank R. Lautenberg Chemical Safety for the 21st Century Act 2016; McCarthy 2016).

Many chemicals have been widely used and then later detected in environmental or biological samples and recognized as hazards to human health (Soto et al. 1991; Rubin 2011; Grandjean and Clapp 2015). A well-known example is the discovery of widespread human exposure and environmental contamination by polybrominated diphenyl ether (PBDE) flame retardants. Swedish researchers characterized the time trend of PBDEs in analyses of breast milk samples (Meironyté et al. 1999), prompting investigations across the globe (Law et al. 2014). PBDEs are structurally similar to polybrominated biphenyl (PBB) flame retardants. PBB flame retardants have been recognized as potentially harmful to human health since the 1970s, after they were inadvertently added to livestock feed in Michigan. In the San Francisco Bay Area, PBDE concentrations approximately 40-fold higher than the Swedish levels were found in marine mammals and human breast adipose tissue samples (She et al. 2002). These unexpected findings prompted policy actions on PBDEs in California and the United States.

In response to the recognition that Californians carried a significant body burden of PBDEs and other toxicants, and the concern that additional exposures to as yet unrecognized chemical hazards were occurring, the California Environmental Contaminant Biomonitoring Program (CECBP)—referred to in this article as Biomonitoring California or the Program—was established by legislation in 2006 (State of California 2006). Biomonitoring California was modeled on the National Biomonitoring Program, which is being implemented by the Centers for Disease Control and Prevention (CDC; http://www.cdc.gov/biomonitoring/about.html). CDC has strongly supported state biomonitoring efforts, providing grants to develop laboratory capability, promote community involvement, and support other aspects of state programs (https://www.cdc.gov/biomonitoring/state_grants.html). Biomonitoring studies at the state level can reveal regional differences in chemical exposures, driven by factors such as product use, types of industry, demographics, and geography. The primary goals of Biomonitoring California are to determine levels of potentially harmful environmental chemicals in the general state population, as well as in sensitive subpopulations (e.g., pregnant women and highly exposed disadvantaged communities); examine time trends in chemical levels; and help assess the effectiveness of public health and regulatory efforts to decrease exposures.

Biomonitoring California is implemented by three California departments: the California Department of Public Health (lead for the overall Program), the Department of Toxic Substances Control, and the Office of Environmental Health Hazard Assessment (OEHHA). The enabling legislation also created the Scientific Guidance Panel (SGP), a body of experts appointed by the Governor and the California State Legislature. The expertise of SGP members encompasses a wide range of disciplines that include epidemiology, toxicology, biostatistics, exposure assessment, laboratory sciences, environmental medicine, public health, maternal and child health, and bioethics. The SGP provides scientific oversight for the design and implementation of Biomonitoring California and formally recommends chemicals to biomonitor. OEHHA convenes the SGP meetings and provides scientific support for the Panel’s chemical selection activities.

For a chemical to be measured in a Biomonitoring California study, it must be on the list of designated chemicals. This list includes all chemicals measured by CDC’s National Biomonitoring Program, as well as chemicals added by a formal vote of the SGP. To inform the SGP’s deliberations, OEHHA researches possible candidate chemicals and develops detailed technical documents on chemicals chosen for consideration. OEHHA’s documents summarize information relevant to the legally mandated criteria for designated chemicals (State of California 2006) that were based on CDC’s selection criteria (CDC 2002) and address the following areas: exposure or potential exposure; known or suspected health effects; analytical factors, such as the availability of a biomonitoring laboratory method; and the need to assess the efficacy of public health actions to reduce exposure. Lack of data on one or more of these criteria does not preclude addition of a chemical to the designated list. The law also specifies criteria that the SGP must follow in identifying priority chemicals, which are chosen from the list of designated chemicals, for biomonitoring. The Program determines which designated or priority chemicals are ultimately biomonitored in specific studies.

In initial efforts to identify chemicals for biomonitoring, the Program invited input from the public via workshops (http://www.biomonitoring.ca.gov/events/workshop-chemical-selection-june-2008), teleconferences, and surveys (CECBP 2009a, 2009b). OEHHA interviewed scientists from a wide range of California agencies, such as those responsible for occupational and public health protection, pesticide regulation, and air and water monitoring, to identify chemicals of greatest concern to state programs. We also consulted with scientists from state, federal, and international biomonitoring programs. Based on this input, an initial pool of chemicals of possible interest for biomonitoring in California was created. OEHHA has added to the pool over time, with a particular focus on chemicals with use patterns likely to increase or decrease as a result of legislative, regulatory, or market actions. For example, following the California ban of most PBDEs, which went into effect in 2006, substitute flame retardants came into much greater use (Dodson et al. 2012; Stapleton et al. 2012). Tracking changing exposures to flame retardants was an early priority for Biomonitoring California, and we have applied this same approach to other chemicals of interest.

The knowledge that PBDE flame retardants were being replaced by numerous compounds, with little information on their market dominance and toxicity, underscored the impracticality of evaluating emerging chemicals individually. In consultation with the SGP and the public, OEHHA shifted toward evaluating groups of chemicals defined by structure and/or function for possible inclusion on Biomonitoring California’s list of designated chemicals. This type of broader and more flexible science-based decision-making is endorsed by the National Research Council (NRC 2009). Our approach has roots in longstanding efforts by other scientists and government agencies to characterize chemical toxicity linked to structural elements (see, for example, European Chemicals Agency 2015). Other agencies have applied approaches similar to OEHHA’s for identifying groups of chemicals, rather than individual chemicals, for various program purposes: U.S. EPA identified a number of flame retardant clusters, such as the chlorinated phosphate ester cluster, for the TSCA Work Plan Chemical Assessment process (https://www.epa.gov/assessing-and-managing-chemicals-under-tsca/fact-sheet-assessing-risks-flame-retardants). The U.S. Food and Drug Administration (FDA 2016) prohibited three perfluoroalkyl ethyl containing substances as indirect food additives, based on structural similarities to chemicals of the same class with toxicity concerns. California’s Safer Consumer Products program employed both functional use categories and structural classes to describe chemicals of potential concern in various product types (DTSC 2015).

In this article, we describe our methods for constructing and researching chemical groups defined by structural features and/or functional uses. We demonstrate the value of our strategy with case studies and discuss unexpected findings.

## Methods

### Overview of Chemical Selection Process

To support the SGP’s chemical selection activities, OEHHA has developed a multi-step process that includes the following: continual tracking of the scientific literature for relevant candidate chemicals; construction of candidate chemical groups; development of preliminary screening information to describe promising candidate chemical groups, which OEHHA presents to the SGP and the public for early input; and development of detailed technical documents on candidate groups chosen for formal consideration. Ultimately, the SGP votes at a public meeting on whether the chemical groups proposed by OEHHA should be included on Biomonitoring California’s list of designated chemicals.

At all stages of this process, OEHHA researches the following main areas:

Structural and functional characteristics of chemical groups and identification of chemicals that are members of those groups.
Exposures or potential exposures, with emphasis on emerging exposures in California.
Potential for toxicity to humans, particularly at doses relevant to environmental exposures.
Ability to biomonitor the chemicals.
Relevance to California statutory and regulatory efforts to reduce chemical exposures.

We carry out our research iteratively, and information uncovered in one of the above areas often prompts us to re-evaluate and refine the chemical groups under consideration.

### Constructing Chemical Groups for Consideration

OEHHA examines our pool of chemicals for common structures and similar uses, evaluates the likelihood of exposures to these chemicals in California, and then constructs logical groups of interest for potential biomonitoring. Some of our guiding principles for defining chemical groups include:

Identify key structural features that are of concern and/or uniquely identify a chemical class. For example:

*p,p*´-Bisphenol structure that is linked to toxicity concerns (Kitamura et al. 2005).
Carbon-fluorine bonds in perfluoroalkyl and polyfluoroalkyl substances (PFASs) that make these chemicals extremely stable and environmentally persistent (OEHHA 2015).
Aim for specificity in capturing important structural elements, but at the same time, define the group broadly enough to avoid excluding important chemicals.
Combine structural characteristics with functional uses to appropriately narrow a very broad group. For example, “brominated compounds” is too broad, but “brominated organic compounds used as flame retardants” is a more manageable group.

Once we define a proposed group, we use an iterative, multi-pronged research strategy to identify chemicals that fall within the group. We use key structural features of the known chemicals in the group to search in databases like PubChem, which has an interface to draw structures. We also search on the various chemical names for those features. For example, “4,4´-bisphenol” and “bis(4-hydroxyphenyl)” are synonyms for “*p,p*´-bisphenol,” and we used all of those terms to find chemicals in this group. Other resources we commonly consult to refine our chemical group include the following databases and documents:

Scientific literature databases, such as PubMed and TOXNET.
Documents by state, federal, and international agencies, such as the National Toxicology Program (NTP) of the National Institute of Environmental Health Sciences and the European Chemicals Agency.
Specialized databases, such as the FDA database on indirect food additives (http://www.fda.gov/Food/IngredientsPackagingLabeling/PackagingFCS/IndirectAdditives/default.htm) and the State of Washington’s Children’s Safe Product Act database (https://fortress.wa.gov/ecy/cspareporting/).
Manufacturers’ websites. Major U.S. manufacturers can be identified using U.S. EPA’s Chemical Data Access Tool (https://java.epa.gov/oppt_chemical_search/).

**Figure 1:**
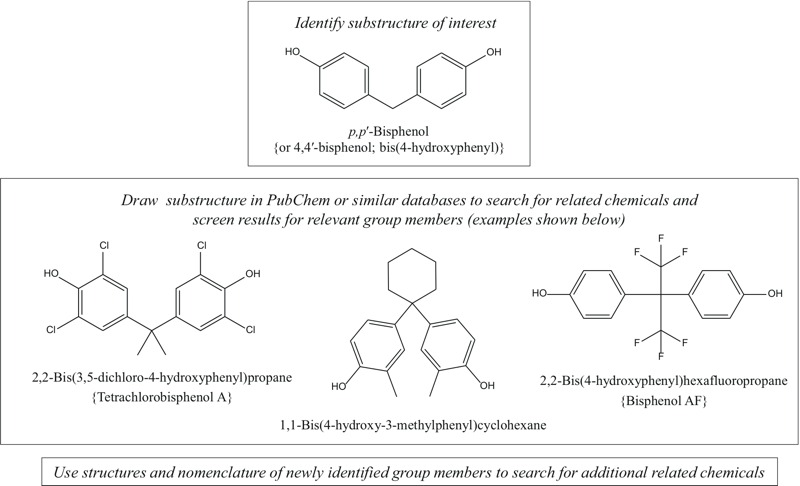
Example search strategy for identifying *p,p*´-bisphenols.

Figure 1 illustrates an example search strategy, which uses a *p,p*´-bisphenol substructure to identify members of this group. The *p,p*´-bisphenol substructure shown has a carbon as the bridging atom; the central atom can also be a sulfur (as in bisphenol S for example).

### Evaluating Exposure or Potential Exposure in California

As one measure of evaluating possible exposure to chemicals in a group, we compile current and past U.S. production/import volume from U.S. EPA’s databases (https://java.epa.gov/oppt_chemical_search/). These data also provide some indication of trends over time.

To locate evidence of current commercial use of chemicals in the group, we also search chemical manufacturers’ websites, including specific product websites; websites of industry associations; and patent applications, which can be accessed in Scopus, for example.

We search literature databases, such as PubMed and TOXNET, using broad terms related to the chemical group (e.g., “brominated flame retardant”) and combine those with search terms related to detections in biological samples, indoor or outdoor environments, and consumer products. These search terms include “exposure”, “biomonitoring”, “urine”, “blood”, “sediment”, “wastewater”, “dust”, and “biota”, and terms for known uses of the chemical (e.g., for bisphenol A [BPA] analogs, known uses include “thermal paper” or “can linings”).

### Researching Known or Suspected Health Effects

In our preliminary screen of chemical groups, we use data from secondary sources, such as documents from national or international agencies, and summaries of toxicity data submitted under the Registration, Evaluation, Authorization, and Restriction of Chemicals (REACH) program. Subsequently, we conduct a detailed literature search for toxicology studies.

Information on known or suspected health effects is often limited for chemical groups of interest. In many cases, we are only able to locate *in vitro* data (including data from high-throughput assays) or *in silico* information (such as structure-activity analyses) for assessing potential toxicity. This type of information can be sufficient to flag a chemical group as posing possible health concerns under our criteria. We focus on evaluating the potential for low-dose effects, which are most relevant to environmental exposures. We search for evidence of subtle biological activity and identify pathways that may be perturbed by that activity. For example, studies indicating effects on hormonal activity or activation of various cellular receptors are of particular interest. We search on key structural features associated with potential toxicity to help identify additional bioactive chemicals and gain insight into possible adverse health effects for the chemical group.

### Assessing Ability to Biomonitor

A practical consideration in choosing chemical groups is determining whether biomarkers for these groups can be detected with sufficient sensitivity in blood or urine samples, which are the biospecimens analyzed by Biomonitoring California. Ideally, the biomarkers would indicate exposure to specific parent compounds versus being common to many different chemicals (e.g., the non-specific dialkyl phosphate metabolites of organophosphate pesticides). We review available metabolism and pharmacokinetic studies, evaluate whether a chemical is likely to be absorbed, and investigate the primary routes of excretion. We also evaluate whether the parent chemicals or their metabolites are likely to be detected via one-time spot sampling in blood or urine, which is the typical biomonitoring study design. Environmentally persistent chemicals and chemicals that bioaccumulate in humans and biota are particularly amenable to one-time biomonitoring. Exposures to short-lived chemicals are more difficult to biomonitor; however, those with continuous exposure patterns (i.e., pseudo-persistent), are good candidates for spot measurements.

As part of our evaluation, we review physical chemical properties that influence the potential for internal exposure to a chemical, including water solubility and log octanol-water partition coefficient (log K_ow_). We also review experimental bioconcentration factors (BCFs) or bioaccumulation factors (BAFs), and half-lives in various environmental media. When experimental values are not available, we use predictive software, such as EPI Suite^TM^ (https://www.epa.gov/tsca-screening-tools/epi-suitetm-estimation-program-interface) to generate values. We use benchmarks from OEHHA’s regulations on Green Chemistry Hazard Traits for California’s Toxics Information Clearinghouse (2012) to flag potentially bioaccumulative and environmentally persistent chemicals (e.g., log K_ow_ ≥ 4 indicates the potential for bioaccumulation).

We also search for past biomonitoring studies that detected chemicals of interest as concrete evidence for the feasibility of biomonitoring.

## Results

OEHHA has applied our strategy, described in the “Methods” section above, to construct and evaluate a wide range of chemical groups. As of July 2015, the SGP has formally reviewed 12 chemical groups proposed by OEHHA and voted to include all of them on the list of designated chemicals. By listing these groups, the SGP has given Biomonitoring California the flexibility to measure any member of these groups in Program studies. After additional research by OEHHA and formal consideration by the SGP, 7 of the 12 groups were added to Biomonitoring California’s list of priority chemicals (Table 1).

**Table 1:** Chemical groups added to Biomonitoring California lists.

Chemical group	Biomonitoring California status		
	Designated chemicals	Priority chemicals	Laboratory capability
Brominated and chlorinated organic compounds used as flame retardants	December 2008	March 2009	Yes
Antimicrobials used in food production	March 2009	—	—
Cyclosiloxanes	March 2009	July 2009	—
Synthetic hormones used in food production	March 2009	—	—
Pyrethroid pesticides	July 2009	—	Yes
Non-halogenated aromatic phosphates	March 2012	April 2013	Yes
*p,p*´*-*Bisphenols	November 2012	April 2013	Yes
Diglycidyl ethers of *p,p*´-bisphenols	November 2012	April 2013	Under development
Polycyclic synthetic musks	November 2013	—	Under development
Tetramethyl acetyloctahydronaphthalenes	November 2013	—	Under development
Perfluoroalkyl and polyfluoroalkyl substances (PFASs)	March 2015	November 2015	Yes
*ortho*-Phthalates	July 2015	November 2015	Yes
Note: The chemical groups are presented in order of listing date. “Yes” means laboratory capability for one or more chemicals in the group.			

The following case studies illustrate our strategy in more detail. We describe the importance of each chemical group in California, how the group was constructed, unexpected findings uncovered through our research, and some challenges in implementing our strategy.

### Flame Retardants

Flame retardants were the first group of chemicals considered as potential designated chemicals. This group was of particular concern in California because of the state’s Technical Bulletin 117 (TB117), a furniture flammability standard that was first put in place in the 1970s by the Bureau of Home Furnishings and Thermal Insulation (BHFTI 2000). TB117 resulted in the extensive use of chemical flame retardants, most notably pentaBDE, the PBDE mixture used in upholstered furniture and other products containing polyurethane foam. In 2008, when the Program began to consider chemicals for possible biomonitoring, California had already banned pentaBDE, but other flame retardants were being used to meet the requirements of TB117 (U.S. EPA 2005). (Note: TB117 was revised effective January 2014, eliminating the need for chemical flame retardants in upholstered furniture purchased in California.)

***Constructing the category.*** Our preliminary screen identified a large number of flame retardants potentially in use or under pre-market development. Most flame retardants that were being detected in the environment and in biota were brominated or chlorinated.

The broad category of “brominated and chlorinated organic chemical compounds used as flame retardants” was constructed based on both structural features and function (OEHHA 2008; 2009). All brominated flame retardants (BFRs) and chlorinated flame retardants (CFRs) that have been adequately studied have shown the potential for toxicity, persistence, and/or bioaccumulation (Birnbaum and Staskal 2004). Constructing this broad group ensured we would capture BFRs and CFRs that might emerge in future market shifts.

Several non-halogenated aromatic phosphate flame retardants were known to be PBDE replacements (Stapleton et al. 2009). Triphenyl phosphate and isopropylated triphenyl phosphate (mixed isomers) were identified as components of Firemaster^®^550, a major PBDE substitute. Because aromatic phosphates are used both as flame retardants and plasticizers, and exposures could occur from either use, we created the structural category of “non-halogenated aromatic phosphates” to encompass all uses (OEHHA 2012a). Although not well studied for toxicity, several aromatic phosphates showed potential for endocrine activity and reproductive toxicity (Honkakoski et al. 2004; Meeker and Stapleton 2010; NTP 1994). At the time of our 2012 review, NTP had announced planned testing of six chemicals in this group, based on a recommendation from the U.S. Consumer Product Safety Commission (NTP 2010). Recently published studies have added to concerns about non-halogenated aromatic phosphates (Behl et al. 2015; Jarema et al. 2015; McGee et al. 2013; Morris et al. 2014).

**Figure 2:**
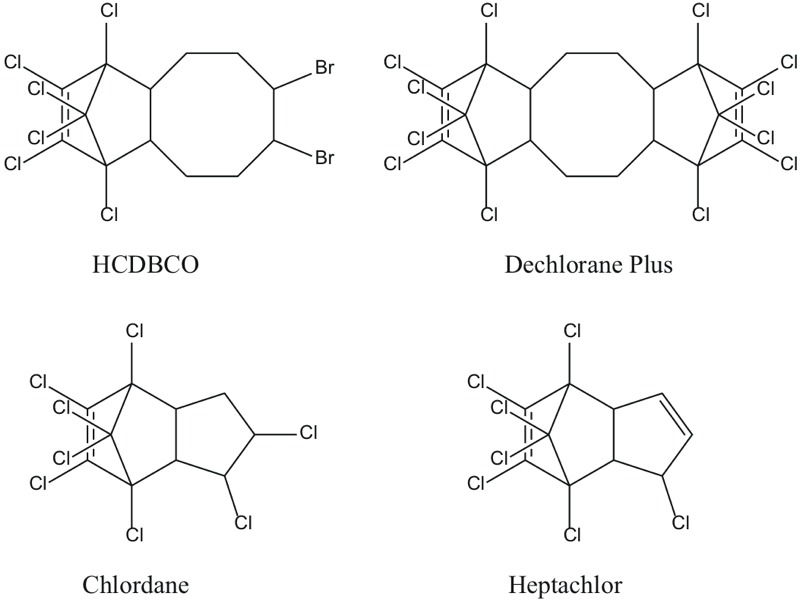
Norbornene rings in halogenated flame retardants and organochlorine pesticides.

***Unexpected findings.*** In evaluating hexachlorocyclopentadienyl-dibromocyclooctane (HCDBCO), a flame retardant with no available toxicity data, we noted a chlorinated norbornene moiety in its structure. Searching on this structural feature, we identified several organochlorine pesticides listed as carcinogens or reproductive toxicants under California’s Proposition 65 (Safe Drinking Water and Toxic Enforcement Act 2013), including dieldrin (cancer), chlordane (cancer), endrin (developmental toxicity), and heptachlor (cancer and developmental toxicity). This structural comparison flagged HCDBCO as a potential health concern, in the absence of toxicity studies. The same norbornene ring is also present in Dechlorane Plus, another flame retardant in this group (Figure 2).

We identified bisphenol A bis(diphenyl phosphate) on a flame retardant manufacturer’s website and then searched the Internet using the CASRN (Chemical Abstracts Service Registry Number) for this chemical. We learned that the Washington State Departments of Health and Ecology (2008) had reviewed it along with another aromatic phosphate, resorcinol bis(diphenyl phosphate), as possible decaBDE substitutes in electronic and plastic consumer products. Searching further, we found that these two aromatic phosphates were part of mixtures, each with a different CASRN, that were reported to have much higher production/import volumes than the individual chemicals (OEHHA 2012a). Recently, both flame retardants have been detected in electronic and consumer products in Europe (Ballesteros-Gómez et al. 2014a) and in dust on electronic equipment at levels up to 1 mg/g dust (Brandsma et al. 2013). This example illustrates the power of an iterative search strategy to identify important members of a chemical group; it also shows how defining a chemical group captures emerging chemicals of potential public health concern.

Some flame retardants we originally thought were emerging actually had substantial U.S. production/import volume for many years (OEHHA 2008, 2009, 2012a). Tris(1,3-dichloro-2-propyl)phosphate (TDCPP), another major pentaBDE substitute, had a production/import volume of 10-50 million pounds every reporting year since 1994, and the structurally related tris(1-chloro-2-propyl)phosphate (TCPP) had a production/import volume of 10-50 million pounds every reporting year since 1990. Isopropylated triphenyl phosphate (mixed isomers) had a long history of high use, with a production/import volume of 10-50 million pounds for every reporting year since 1986. Triphenyl phosphate had the same high volume for every reporting year since 1998. Due to a lack of toxicity and exposure information, these flame retardants had not previously come to regulatory attention.

***Challenges in implementing our strategy.*** Because chemical manufacturers can withhold the identity of chemicals as confidential business information (CBI), we were limited in our ability to identify key chemicals in these classes. In an assessment of potential pentaBDE substitutes, U.S. EPA indicated that 12 of the 15 potential substitutes were proprietary, and thus did not provide the chemical names or CASRNs in their report (U.S. EPA 2005). We also found that using U.S. production/import volume as an indicator of exposure has significant limitations: U.S. EPA collects this information only every four years, so it is frequently out of date. Companies can also claim production/import volume as CBI, and these data are then withheld from U.S. EPA’s public database.

### p,p*´*-Bisphenols and Diglycidyl Ethers of p,p*´*-Bisphenols

The California ban on BPA in baby bottles and sippy cups, which went into effect in 2013 (Product Safety: Bisphenol A 2011), along with similar initiatives in other jurisdictions, spurred the development of BPA substitutes for plastics. Alternatives to BPA were also being developed for use in thermal paper and for food and beverage can linings. Prompted by growing potential health concerns associated with known BPA substitutes, such as bisphenol S (BPS), the Program conducted a preliminary screen as the first step in identifying emerging substitutes to biomonitor (OEHHA 2012b).

***Constructing the category.*** Our preliminary screen reviewed 23 substances that were potential BPA substitutes and/or structural analogs (OEHHA 2012b). In several experimental systems, the estrogenicity and/or anti-androgenicity of some *p,p*´-bisphenol analogs was of the same order of magnitude or greater than that of BPA (Kitamura et al. 2005; Matsushima et al. 2010; Zhang et al. 2011). These findings have been confirmed in recent studies (Eladak et al. 2015; Rochester and Bolden 2015).

Based on the results of our preliminary screen, the SGP advised OEHHA to focus first on chemicals with the key structural features of BPA, rather than the structurally diverse category of “BPA substitutes.” We therefore chose the structural groups “*p*,*p*´-bisphenols” and “diglycidyl ethers of *p*,*p*´-bisphenols” for in depth research (OEHHA 2012c). The chemicals in these groups contain either a *p*-hydroxy (*p-*OH) moiety on each aromatic ring (*p*,*p*´-bisphenols) or an epoxypropyl ether moiety in place of the hydroxy group (diglycidyl ethers).

***Unexpected findings.*** Of the large number of *p,p*´-bisphenols we identified, very few were in U.S. EPA’s public database on production/import volume. Our search strategy of industry websites proved particularly effective in verifying the use of certain bisphenols for which U.S. production/import volume data were not available. For example, no production/import volume was reported in U.S. EPA’s database for either bisphenol F (BPF) or bisphenol F diglycidyl ether (BFDGE) from 1986 through 2006. However, from a chemical manufacturer’s website, we identified a BFDGE-based epoxy resin that was marketed as compliant with FDA regulations for food contact applications (OEHHA 2012c). The resin is formed via a reaction between BPF and epichlorohydrin and has its own CASRN, under which U.S. production/import volume was reported. We located a second epoxy resin, also with its own separate CASRN, that is formed from the mixture of BPA and BPF and epichlorohydrin. Liao et al. (2012) found BPF in more than 65% of indoor dust samples (*n* = 38) in Albany, New York, verifying potential human exposure to this chemical.

### Synthetic Musks and Tetramethylacetyl Octahydronaphthalenes

Synthetic musk fragrance compounds are common ingredients in a variety of cosmetics and personal care products. Nitromusks and polycyclic musks have been detected in biomonitoring studies primarily in Europe and Asia. We initially screened four classes of synthetic musks: nitromusks, and polycyclic, macrocyclic and alicyclic musks. The screen showed that the use of nitromusks has been declining since the 1980s. Three nitromusks (musk moskene, musk tibetene, and musk ambrette) had been discontinued or prohibited because of potential health concerns. Musk xylene, one of two nitromusks still in use (musk ketone is the other) has been identified as a Substance of Very High Concern (SVHC) in the European Union due to its very persistent and very bioaccumulative (vP/vB) properties (European Commission 2011).

We found that the decline in the use of nitromusks occurred in parallel with a marked increase in use of polycyclic musks. Widespread exposure to polycyclic musks has been demonstrated via detections in blood, adipose tissue, and breast milk (OEHHA 2013a). More recently, the use of polycyclic musks has been declining in Europe, but this does not appear to be the case in North America. At the same time, macrocyclic musks have come into greater use in Europe and in North America. Low levels of one macrocyclic musk were found in house dust samples from a Canadian study (Kubwabo et al. 2012). Alicyclic musks appeared to be in use at the time we conducted our preliminary screen, but we found very little information on exposure or toxicity. We also identified a structurally related fragrance compound commonly referred to as OTNE 1-(1,2,3,4,5,6,7,8-octahydro-2,3,8,8-tetramethyl-2-naphthalenyl)ethanone that had been detected in dust (Kubwabo et al. 2012).

***Constructing the categories.*** Based on our preliminary screen, the SGP advised OEHHA to conduct a review of all four categories of musks, as well as OTNE. In further research, OEHHA determined that musk xylene had been prohibited by the International Fragrance Association (IFRA 2009) and that use of musk ketone had sharply declined in North America. We did not locate adequate toxicity or exposure information for macrocyclic or alicyclic musks. Thus, for our in depth review, we focused on synthetic polycyclic musks and the structurally related compound OTNE.

Synthetic polycyclic musks were still in widespread use, and we had evidence of exposure and toxicity concerns (OEHHA 2013a). OTNE was a high production volume chemical with exposure potential demonstrated by detections in dust. In searching for various possible chemical names for OTNE, we uncovered three additional isomers that were also used in fragrances. Based on these findings, we developed the structural category “tetramethyl acetyloctahydronaphthalenes,” which encompassed OTNE and the three additional isomers (OEHHA 2013b).

***Unexpected findings.*** After constructing our fragrance compound categories, we found that the four tetramethyl acetyloctahydronaphthalenes were each flagged by U.S. EPA (2012) as TSCA Workplan Chemicals, based on aquatic toxicity and the potential for human exposure.

## Discussion

Determining which chemicals are important to prioritize for biomonitoring and other environmental and public health efforts is a continual topic of research and discussion. Production/import volume is often relied upon as an indicator of exposure, but these data have been limited by infrequent reporting and CBI claims. As of 2010, 19% of the 84,000 chemicals in the TSCA Inventory were classified as confidential (Goodman 2010). U.S. EPA has reserved the right to review and potentially reject claims of confidentiality, though CBI claims were typically honored as long as procedural requirements were met (https://www.epa.gov/tsca-cbi/about-confidential-business-information-cbi-claims-and-their-reviews-under-tsca). Recent legislation has increased the requirements for a valid CBI claim (Frank R. Lautenberg Chemical Safety for the 21st Century Act 2016). As an additional impediment to identifying chemicals in use, manufacturers are not required to disclose ingredients of many consumer products. Further, there are no data on the volume of chemicals that enter the United States as part of finished products imported from other countries.

Even with the best possible data on use and production/import volume, this information is not equivalent to exposure data. Detection in indoor dust, such as house and office dust, can be a good indicator of current use and exposure for certain types of contaminants. Dust is simpler to collect than blood or urine, and contaminant levels in dust are generally much higher than concentrations of biomarkers in biological samples. Finding chemicals of potential concern in dust first can confirm the importance of these for human biomonitoring. However, most dust studies use targeted measurement approaches, which require that the chemicals are already known and considered worth measuring. Further, for emerging chemicals, analytical standards may not be commercially available.

Non-targeted and semi-targeted screening of environmental media and biological samples for a wide array of contaminants is a promising way to identify previously undetected chemicals (Ballesteros-Gómez et al. 2014b; Crimmins et al. 2014; Jamin et al. 2014; Hilton et al. 2010). This method includes the application of specialized mass spectrometry techniques that accurately measure molecular masses, which are then used to establish plausible chemical identities. Crimmins et al. (2014) used non-targeted search strategies to tentatively identify a novel perfluorinated compound in Lake Ontario trout. Ballesteros-Gómez et al. (2014b) identified a previously unknown BFR in plastic consumer products using non-targeted screening and then later targeted and found the same new BFR in house dust. Our laboratories are currently developing non-targeted methods, which have the potential to dramatically expand the scope and effectiveness of our strategy to identify and prioritize chemicals for biomonitoring.

The lack of toxicological data for many chemicals remains a major challenge for prioritization efforts. New high-throughput toxicity screening projects (e.g., ToxCast™ and Tox21) are beginning to address this data gap (Attene-Ramos et al. 2013; Kavlock et al. 2012). These innovative toxicity data hold promise for ultimately identifying structural features that predict toxicity potential.

## Conclusion

We construct broad functional and structural chemical groups to proactively and efficiently capture chemicals of potential concern for inclusion in Biomonitoring California. These groups encompass chemicals already in high use, as well as related chemicals that may later emerge as exposure and health concerns. Listing of broad groups allows us to keep up with market shifts and respond to other new information in choosing chemicals to measure. The chemical selection strategy described in this paper can be applied in other monitoring, testing, and assessment programs to support early action on emerging chemicals. Ultimately, taking a more comprehensive approach to identifying chemicals with potential exposure and toxicity concerns will improve the protection of public health and the environment.

## References

[r1] Attene-Ramos MS, Miller N, Huang R, Michael S, Itkin M, Kavlock RJ (2013). The Tox21 robotic platform for the assessment of environmental chemicals—from vision to reality.. Drug Discov Today.

[r2] Ballesteros-Gómez A Brandsma SH de Boer J Leonards PE 2014a Analysis of two alternative organophosphorus flame retardants in electronic and plastic consumer products: resorcinol bis-(diphenylphosphate) (RBDPP) and bisphenol A bis (diphenylphosphate) (BPA-BDPP). Chemosphere 116 10 14, doi:10.1016/j.chemosphere.2013.12.099 24556545

[r3] Ballesteros-Gómez A, de Boer J, Leonards P (2014b). A novel brominated triazine-based flame retardant (TTBP-TAZ) in plastic consumer products and indoor dust.. Environ Sci Technol.

[r4] Behl M, Hsieh JH, Shafer TJ, Mundy WR, Rice JR, Boyd WA, et al (2015). Use of alternative assays to identify and prioritize organophosphorus flame retardants for potential developmental and neurotoxicity.. Neurotoxicol Teratol.

[r5] BHFTI (Bureau of Home Furnishings and Thermal Insulation) (2000). Technical Bulletin 117. Requirements, Test Procedure and Apparatus for Testing the Flame Retardance of Resilient Filling Materials Used in Upholstered Furniture.. http://www.bearhfti.ca.gov/industry/117.pdf.

[r6] Birnbaum LS, Staskal DF (2004). Brominated flame retardants: cause for concern?. Environ Health Perspect.

[r7] Brandsma SH, Sellström U, de Wit CA, de Boer J, Leonards PE (2013). Dust measurement of two organophosphorus flame retardants, resorcinol bis(diphenylphosphate (RBDPP) and bisphenol a bis(diphenylphosphate) (BPA-BDPP), used as alternatives to BDE-209.. Environ Sci Technol.

[r8] CDC (Centers for Disease Control and Prevention) (2002). Final selection criteria and solicitation of nominations for chemicals or categories of environmental chemicals for analytic development and inclusion in future releases of the National Report on Human Exposure to Environmental Chemicals.. Fed Reg.

[r9] CECBP (California Environmental Contaminant Biomonitoring Program) (2009a). Results of Public Participation Activities on What Chemicals Should be Biomonitored in California. A Report of the California Environmental Contaminant Biomonitoring Program.. http://www.biomonitoring.ca.gov/sites/default/files/downloads/PublicParticipationreport021909.pdf.

[r10] CECBP (2009b). Staff Responses to State Government Query on Chemicals for Biomonitoring. A Report of the California Environmental Contaminant Biomonitoring Program.. http://www.biomonitoring.ca.gov/sites/default/files/downloads/StateGovReport021909.pdf.

[r11] Crimmins BS, Xia X, Hopke PK, Holsen TM (2014). A targeted/non-targeted screening method for perfluoroalkyl carboxylic acids and sulfonates in whole fish using quadrupole time-of-flight mass spectrometry and MSe.. Anal Bioanal Chem.

[r12] Dodson RE, Perovich LJ, Covaci A, Van den Eede N, Ionas AC, Dirtu AC (2012). After the PBDE phase-out: a broad suite of flame retardants in repeat house dust samples from California.. Environ Sci Technol.

[r13] DTSC (California Department of Toxic Substances Control) (2015). Priority Product Work Plan. Three Year Work Plan 2015-2017.. https://www.dtsc.ca.gov/SCP/upload/PriorityProductWorkPlan_2015.pdf.

[r14] Egeghy PP Judson R Gangwal S Mosher S Smith D Vail J 2012 The exposure data landscape for manufactured chemicals. Sci Total Environ 414 159 166, doi:10.1016/j.scitotenv.2011.10.046 22104386

[r15] Eladak S, Grisin T, Moison D, Guerquin MJ, N’Tumba-Byn T, Pozzi-Gaudin S (2015). A new chapter in the bisphenol A story: bisphenol S and bisphenol F are not safe alternatives to this compound.. Fertil Steril.

[r16] European Chemicals Agency (2015). Read-Across Assessment Framework (RAAF).. http://echa.europa.eu/documents/10162/13628/raaf_en.pdf.

[r17] European Commission (2011). Commission Regulation (EU) No. 143/2011 of 17 February 2011. Amending Annex XIV to Regulation (EC) No. 1907/2006 of the European Parliament and of the Council on the Registration, Evaluation, Authorisation and Restriction of Chemicals (REACH).. Official J Eur Union 18.2.2011.

[r18] FDA (U.S. Food and Drug Administration) (2016). Indirect Food Additives: Paper and Paperboard Components. Final Rule. January 4, 2016.. https://www.gpo.gov/fdsys/pkg/FR-2016-01-04/pdf/2015-33026.pdf.

[r19] Frank R. Lautenberg Chemical Safety for the 21st Century Act (2016). Public Law 114-182, June 22, 2016.. https://www.congress.gov/bill/114th-congress/house-bill/2576/text.

[r20] GAO (Government Accountability Office) (2013). High-Risk Series - An Update. Report to Congressional Committees. Chapter on Transforming EPA’s Processes for Assessing and Controlling Toxic Chemicals. pp. 209-212.. http://www.gao.gov/assets/660/652133.pdf.

[r21] Goodman S (2010). States push EPA, Congress to curb business confidentiality claims for chemicals [news story].. *New York Times*. Energy & Environment, 1 March 2010.

[r22] Grandjean P, Clapp R (2015). Perfluorinated alkyl substances: emerging insights into health risks.. New Solut.

[r23] Green Chemistry Hazard Traits for California’s Toxics Information Clearinghouse (2012). California Code of Regulations, Title 22, Division 4.5, Chapter 54, §§ 69401–69407.2.. http://oehha.ca.gov/media/downloads/risk-assessment//gcregtext011912.pdf.

[r24] Hilton DC, Jones RS, Sjödin A (2010). A method for rapid, non-targeted screening for environmental contaminants in household dust.. J Chromatogr A.

[r25] Honkakoski P, Palvimo JJ, Penttilä L, Vepsäläinen J, Auriola S (2004). Effects of triaryl phosphates on mouse and human nuclear receptors.. Biochem Pharmacol.

[r26] IFRA (International Fragrance Association) (2009). Musk Xylene. IFRA Standard.. 44th Amendment.

[r27] Jamin EL, Bonvallot N, Tremblay-Franco M, Cravedi JP, Chevrier C, Cordier S (2014). Untargeted profiling of pesticide metabolites by LC–HRMS: an exposomics tool for human exposure evaluation.. Anal Bioanal Chem.

[r28] Jarema KA, Hunter DL, Shaffer RM, Behl M, Padilla S (2015). Acute and developmental behavioral effects of flame retardants and related chemicals in zebrafish.. Neurotoxicol Teratol.

[r29] Judson R Richard A Dix DJ Houck K Martin M Kavlock R 2009 The toxicity data landscape for environmental chemicals. Environ Health Perspect 117 5 685 695, doi:10.1289/ehp.0800168 19479008 PMC2685828

[r30] Kavlock R, Chandler K, Houck K, Hunter S, Judson R, Kleinstreuer N (2012). Update on EPA’s ToxCast program: providing high throughput decision support tools for chemical risk management.. Chem Res Toxicol.

[r31] Kitamura S, Suzuki T, Sanoh S, Kohta R, Jinno N, Sugihara K (2005). Comparative study of the endocrine-disrupting activity of bisphenol A and 19 related compounds.. Toxicol Sci.

[r32] Kubwabo C, Fan X, Rasmussen P, Wu F (2012). Determination of synthetic musk compounds in indoor house dust by gas chromatography–ion trap mass spectrometry.. Anal Bioanal Chem.

[r33] Law RJ, Covaci A, Harrad S, Herzke D, Abdallah MA, Fernie K (2014). Levels and trends of PBDEs and HBCDs in the global environment: status at the end of 2012.. Environ Int.

[r34] Liao C, Liu F, Guo Y, Moon HB, Nakata H, Wu Q (2012). Occurrence of eight bisphenol analogues in indoor dust from the United States and several Asian countries: implications for human exposure.. Environ Sci Technol.

[r35] Matsushima A Liu X Okada H Shimohigashi M Shimohigashi Y 2010 Bisphenol AF is a full agonist for the estrogen receptor ERα but a highly specific antagonist for Erβ. Environ Health Perspect 118 1267 1272, doi:10.1289/ehp.0901819 20427257 PMC2944088

[r36] McCarthy G (2016). TSCA reform: a bipartisan milestone to protect our health from dangerous chemicals [press release].. *EPA Connect*, 22 June 2016.

[r37] McGee S, Konstantinov A, Stapleton HM, Volz D (2013). Aryl phosphate esters within a major pentaBDE replacement product induce cardiotoxicity in developing zebrafish embryos: potential role of the aryl hydrocarbon receptor.. Toxicol Sci.

[r38] Meeker JD Stapleton HM 2010 House dust concentrations of organophosphate flame retardants in relation to hormone levels and semen quality parameters. Environ Health Perspect 118 3 318 323, doi:10.1289/ehp.0901332 20194068 PMC2854757

[r39] Meironyté D, Noren K, Bergman A (1999). Analysis of polybrominated diphenyl ethers in Swedish human milk. A time-related trend study, 1972–1997.. J Toxicol Environ Health A.

[r40] Morris PJ, Medina-Cleghorn D, Heslin A, King SM, Orr J, Mulvihill MM (2014). Organophosphorus flame retardants inhibit specific liver carboxylesterases and cause serum hypertriglyceridemia.. ACS Chem Biol.

[r41] Neltner TG Alger HM Leonard JE Maffini MV 2013 Data gaps in toxicity testing of chemicals allowed in food in the United States. Reprod Toxicol 42 85 94, doi:10.1016/j.reprotox.2013.07.023 23954440

[r42] NRC (National Research Council) (2009). Science and Decisions: Advancing Risk Assessment..

[r43] NTP (National Toxicology Program) (1994). Toxicology and Carcinogenesis Studies of Tricresyl Phosphate (CAS No. 1330-78-5) in F344/N Rats and B6C3F1 Mice (Gavage and Feed Studies).. Technical Report Series No. 433.

[r44] NTP (2010). TP Research Concept Update: Selected Flame Retardants. Summary Minutes, NTP Board of Scientific Counselors Meeting – 30 November–1 December 2010.. http://ntp.niehs.nih.gov/ntp/about_ntp/bsc/2010/novdec/minutes20101201.pdf.

[r45] OEHHA (Office of Environmental Health Hazard Assessment) (2008). Brominated and Chlorinated Organic Chemical Compounds Used as Flame Retardants. Consideration of Potential Designated Chemicals.. http://www.biomonitoring.ca.gov/sites/default/files/downloads/120408flamedoc.pdf.

[r46] OEHHA (2009). Brominated and Chlorinated Organic Chemical Compounds Used as Flame Retardants. Additional Information on Four Flame Retardants.. http://www.biomonitoring.ca.gov/sites/default/files/downloads/FlameRetardants_FourMore.pdf.

[r47] OEHHA (2012a). Non-halogenated Aromatic Phosphates. Potential Designated Chemicals.. http://www.biomonitoring.ca.gov/sites/default/files/downloads/031612NhArPvers3.pdf.

[r48] OEHHA (2012b). Preliminary Screen for Possible Future Consideration as Potential Designated Chemicals for Biomonitoring California. Some Bisphenol A Substitutes and Structurally Related Compounds.. http://biomonitoring.ca.gov/sites/default/files/downloads/031612PrelimScreen2.pdf.

[r49] OEHHA (2012c). *p,p*´-Bisphenols and Diglycidyl Ethers of *p,p*´-Bisphenols. Potential Designated Chemicals.. http://www.biomonitoring.ca.gov/sites/default/files/downloads/110812Bisphenols.pdf.

[r50] OEHHA (2013a). Synthetic Polycyclic Musks. Potential Designated Chemicals.. http://www.biomonitoring.ca.gov/sites/default/files/downloads/110813PolycyclicMusksDesig3.pdf.

[r51] OEHHA (2013b). Tetramethyl Acetyloctahydronaphthalenes. Potential Designated Chemicals.. http://www.biomonitoring.ca.gov/sites/default/files/downloads/110113TetramethylAcetyloctahydronaphthalenesDesig.pdf.

[r52] OEHHA (2015). Perfluoroalkyl and Polyfluoroalkyl Substances (PFASs). Potential Designated Chemicals.. http://biomonitoring.ca.gov/sites/default/files/downloads/PotenDesigPFASs_031315.pdf.

[r53] Product Safety: Bisphenol A (2011). California Health and Safety Code, Division 104, Part 3, Chapter 11, §§ 108940–108941.

[r54] Rochester JR Bolden AL 2015 Bisphenol S and F: A systematic review and comparison of the hormonal activity of bisphenol A substitutes. Environ Health Perspect 123 7 643 650, doi:10.1289/ehp.1408989 25775505 PMC4492270

[r55] Rubin BS (2011). Bisphenol A: an endocrine disruptor with widespread exposure and multiple effects.. J Steroid Biochem Mol Biol.

[r56] Safe Drinking Water and Toxic Enforcement Act (2013). California Health and Safety Code, Division 20, Chapter 6.6, §§ 25249.5–25249.13.. Passed as Proposition 65 in 1986, amended in 2013.

[r57] She J, Petreas M, Winkler J, Visita P, McKinney M, Kopec D (2002). PBDEs in the San Francisco Bay Area: measurement in harbor seal blubber and human breast adipose tissue.. Chemosphere.

[r58] Silbergeld EK, Mandrioli D, Cranor CF (2015). Regulating chemicals: law, science, and the unbearable burdens of regulation.. Ann Rev Pub Health.

[r59] Soto AM, Justicia H, Wray JW, Sonnenschein C (1991). *p*-Nonyl-phenol: an estrogenic xenobiotic released from “modified” polystyrene.. Environ Health Perspect.

[r60] Stapleton HM, Klosterhaus S, Eagle S, Fuh J, Meeker JD, Blum A (2009). Detection of organophosphate flame retardants in furniture foam and U.S. house dust.. Environ Sci Technol.

[r61] Stapleton HM, Sharma S, Getzinger G, Ferguson P, Gabriel M, Webster T (2012). Novel and high volume use flame retardants in U.S. couches reflective of the 2005 pentaBDE phase out.. Environ Sci Technol.

[r62] State of California (2006). California Environmental Contaminant Biomonitoring Program.. California Health and Safety Code, Division 103, Part 5, Chapter 8, §§ 105440-105459 (September 29, 2006).

[r63] U.S. EPA (U.S. Environmental Protection Agency) (2005). Furniture Flame Retardancy Partnership. Environmental Profiles of Chemical Flame-Retardant Alternatives for Low-Density Polyurethane Foam. Volume One. EPA 742-R-05-002A. September 2005.. https://www.epa.gov/sites/production/files/2015-04/documents/ffr_foam_alternatives_vol1.pdf.

[r64] U.S. EPA (2012). TSCA Work Plan Chemicals.. https://www.epa.gov/sites/production/files/2014-02/documents/work_plan_chemicals_web_final.pdf.

[r65] Washington State Departments of Health and Ecology (2008). Alternatives to Deca-BDE in Televisions and Computers and Residential Upholstered Furniture. Final Report. December 29, 2008.. Department of Health Publication No. 334-181. Department of Ecology No. 09-07-041.

[r66] Zhang HC, Hu XL, Yin DQ, Lin ZF (2011). Development of molecular docking-based binding energy to predict the joint effect of BPA and its analogs.. Hum Exp Toxicol.

